# Increase in invasive group A streptococcal infections in Milan, Italy: a genomic and clinical characterization

**DOI:** 10.3389/fmicb.2023.1287522

**Published:** 2024-01-11

**Authors:** Davide Mangioni, Valeria Fox, Paola Saltini, Andrea Lombardi, Linda Bussini, Francesco Carella, Lisa Cariani, Agnese Comelli, Caterina Matinato, Antonio Muscatello, Antonio Teri, Leonardo Terranova, Valeria Cento, Sara Carloni, Michele Bartoletti, Claudia Alteri, Alessandra Bandera

**Affiliations:** ^1^Infectious Diseases Unit, Foundation Istituto di Ricovero e Cura a Carattere Scientifico (IRCCS) Ca' Granda Ospedale Maggiore Policlinico, Milan, Italy; ^2^Department of Pathophysiology and Transplantation, University of Milano, Milan, Italy; ^3^Department of Oncology and Hemato-Oncology, University of Milan, Milan, Italy; ^4^Infectious Disease Unit, Istituto di Ricovero e Cura a Carattere Scientifico (IRCCS) Humanitas Research Hospital, Milan, Italy; ^5^Department of Biomedical Sciences, Humanitas University, Milan, Italy; ^6^Microbiology Laboratory, Clinical Pathology, Foundation Istituto di Ricovero e Cura a Carattere Scientifico (IRCCS) Ca' Granda Ospedale Maggiore Policlinico, Milan, Italy; ^7^Respiratory Unit and Adult Cystic Fibrosis Center, Department of Internal Medicine, Foundation Istituto di Ricovero e Cura a Carattere Scientifico (IRCCS) Cà Granda Ospedale Maggiore Policlinico, Milan, Italy; ^8^Microbiology and Virology, Istituto di Ricovero e Cura a Carattere Scientifico (IRCCS) Humanitas Research Hospital, Milan, Italy; ^9^Istituto di Ricovero e Cura a Carattere Scientifico (IRCCS) Humanitas Research Hospital, Rozzano, Italy

**Keywords:** *Streptococcus pyogenes*, GAS, necrotizing fasciitis, virulence factors, WGS, genomic surveillance

## Abstract

**Background:**

Group A Streptococcus (GAS) causes multiple clinical manifestations, including invasive (iGAS) or even life-threatening (severe-iGAS) infections. After the drop in cases during COVID-19 pandemic, in 2022 a sharp increase of GAS was reported globally.

**Methods:**

GAS strains collected in 09/2022–03/2023 in two university hospitals in Milan, Italy were retrospectively analyzed. Clinical/epidemiological data were combined with whole-genome sequencing to: (i) define resistome/virulome, (ii) identify putative transmission chains, (iii) explore associations between *emm-*types and clinical severity.

**Results:**

Twenty-eight isolates were available, 19/28 (67.9%) from adults and 9/28 (32.1%) from pediatric population. The criteria for iGAS were met by 19/28 cases (67.9%), of which 11/19 (39.3%) met the further criteria for severe-iGAS. Pediatric cases were mainly non-invasive infections (8/9, 88.9%), adult cases were iGAS and severe-iGAS in 18/19 (94.7%) and 10/19 (52.6%), respectively. Thirteen *emm*-types were detected, the most prevalent being *emm*1 and *emm*12 (6/28 strains each, 21.4%). Single nucleotide polymorphism (SNP) analysis of *emm*1.0 and *emm*12.0 strains revealed pairwise SNP distance always >10, inconsistent with unique transmission chains. *Emm*12.0-type, found to almost exclusively carry virulence factors *spe*H and *spe*I, was mainly detected in children and in no-iGAS infections (55.6 vs. 5.3%, *p* = 0.007 and 66.7 vs. 0.0%, *p* < 0.001, respectively), while *emm*1.0-type was mainly detected in severe-iGAS (0.0 vs. 45.5%, *p* = 0.045).

**Conclusions:**

This study showed that multiple *emm*-types contributed to a 2022/2023 GAS infection increase in two hospitals in Milan, with no evidence of direct transmission chains. Specific *emm*-types could be associated with disease severity or invasiveness. Overall, these results support the integration of classical epidemiological studies with genomic investigation to appropriately manage severe infections and improve surveillance.

## Introduction

*Streptococcus pyogenes* (Group A Streptococcus, GAS) is a human-specific Gram-positive coccus responsible for a broad spectrum of diseases differing in clinical presentation and severity (Brouwer et al., 2023). Primary infection sites are usually upper airways and skin, where non-invasive GAS infections occur and from where the pathogen can be transmitted to a new host or can disseminate causing invasive disease (iGAS) such as empyema (infection of the pleural cavity), streptococcal toxic shock syndrome (STSS) or necrotising fasciitis (Efstratiou and Lamagni, 2022; Stevens and Bryant, 2022a,b; GOV.UK, 2023). iGAS infections can rapidly progress and often require prompt surgical treatment to obtain rapid and adequate source control (Stevens et al., 2014; Johnson and LaRock, 2021). They are also characterized by high morbidity and mortality rates, with ~8–23% of deaths within 7 days from infection (Walker et al., 2014). Incidence of GAS diseases varies with season and geographical location. Yet, while outbreaks of non-invasive infections are fairly common in occurence, iGAS are fortunately rare conditions (Efstratiou and Lamagni, 2022; Brouwer et al., 2023).

The mechanisms of GAS infection are complex and multifactorial, related to a large number of virulence determinants. A major virulence factor is represented by the M-protein, a surface protein which forms fibrils on the surface of the bacteria (Ghosh, 2011), encoded by the *emm* gene and basis of the most widely used method of epidemiological genotyping (Beall et al., 1996; Facklam et al., 1999). Certain *emm* types are linked to a higher risk of developing worse clinical manifestations, such as e*mm*1, which has been related to iGAS cases including necrotizing fasciitis and STSS (Friães et al., 2012). Additional important virulence factors include T-cell superantigens, which are able to cross-link the major histocompatibility complex (MHC) class II with the T-cell receptor (TCR) on antigen presenting cells inducing extensive T-cell activation and proliferation contributing to pathologies such as streptococcal toxic shock syndrome (Proft and Fraser, 2022). GAS carry various superantigens, some found on the chromosome, and some associated with prophages (McShan and Nguyen, 2022).

In late 2022, several countries of the European Region and the United Kingdom reported a marked increase of scarlet fever and iGAS infections, mostly affecting children under 10 years of age (ECDC, 2022; GOV.UK, 2022; WHO, 2022). This was presumed to be caused by an early start of the GAS infection season together with lowered immunity following 2 years of strict social distancing and reduced GAS infections during COVID-19 pandemic (de Gier et al., 2019, 2023; GOV.UK, 2022; Lassoued et al., 2023). This surge in infections where investigated was found to be caused by GAS of multiple *emm*-types, and in general was not dominated by the emergence and expansion of a single clone. This sharp increase of GAS infections seems not determined by specific new *emm*-type or obvious expansion of a single clone (de Gier et al., 2023; Guy et al., 2023).

Between the last quarter of 2022 and the 1st months of 2023, an increased incidence of iGAS infections was observed in our hospitals in the Milan area (Italy), especially in adult patients. To provide exploratory information regarding epidemiological, clinical, and microbial characteristics of GAS infections during this timeframe, we conducted this retrospective, observational study on cases with bacterial strain available for genomic characterization. To provide background information on incidence of GAS infections in the two centers, we also calculated the temporal trend of laboratory-identified cases during the preceding 7 years.

## Methods

### Study setting and population

All the available bacterial strains of *Streptococcus pyogenes* at Fondazione IRCCS Ca' Granda Ospedale Maggiore Policlinico of Milan and IRCCS Humanitas Research Hospital collected from September 2022 to March 2023 were included. The two centers are large university hospitals located in Milan, Northern Italy. For the period investigated, Fondazione IRCCS Ca' Granda Ospedale Maggiore Policlinico of Milan archived *as for* clinical practice bacteria strains from both invasive cases (blood cultures, intraoperative cultures) and non-invasive infections (pharyngeal swabs, nasopharyngeal aspirates), and for IRCCS Humanitas Research Hospital only blood culture isolates were obtained.

Clinical and epidemiological data were retrospectively collected from hospital records and databases.

Patients below 18 years of age were considered pediatric cases. Non-invasive GAS (non-iGAS) infections and iGAS infections were defined based on microbiological and clinical criteria according to literature (Stevens and Bryant, 2022a,b; GOV.UK, 2023). Non-iGAS infections included upper airway respiratory infections, scarlet fever, and superficial acute bacterial skin and skin structure infections (ABSSSIs; Stevens and Bryant, 2022a). iGAS infections included cases with isolation of *Streptococcus pyogenes* from normally sterile body sites as well as severe clinical presentations caused by GAS even if not isolated from a normally sterile material (Stevens and Bryant, 2022b; GOV.UK, 2023). Within iGAS group, we defined as severe iGAS the life-threatening conditions associated with the highest mortality such as sepsis/septic shock, necrotizing fasciitis and STSS (Stevens and Bryant, 2022b). Temporal trends of culture-confirmed non-iGAS and iGAS cases were calculated from 2015 to the first quarter of 2023.

### Antimicrobial susceptibility testing

Antimicrobial susceptibility testing after MALDI-ToF identification (VITEK-MS, bioMérieux or Bruker Diagnostics) was performed by automated systems (VITEK2 compact automated system, bioMérieux or Phoenix automated microbiology system, Becton Dickinson Diagnostic Systems). Susceptible, intermediate and resistant categories were assigned according to the EUCAST breakpoint table (version 13.0, available at https://www.eucast.org/clinical_breakpoints).

### Whole genome sequencing analysis and bacterial typing

Genomic DNA was extracted from pure isolates using ZymoBIOMICS DNA Miniprep Kit (Zymo Reseach) in accordance with manufacturer's instructions. Libraries for whole genome sequencing were generated using Illumina DNA Library Prep kit (Illumina, Inc., San Diego, CA, USA) and sequenced on Illumina MiSeq sequencing platform (Illumina, San Diego, CA, USA) using MiSeq Reagent Kit v2 to obtain 150-bp paired-end reads. Raw reads were trimmed for adapters and filtered for quality (average phred score>20) with Fastp (v0.20.1) and quality checked after trimming with FastQC (v0.11.9; Andrews, 2010; Chen et al., 2018). Kraken (v1.1.1) was used to screen for potential contaminations (Wood and Salzberg, 2014). Whole genome sequencing reads were assembled *de novo* using SPAdes (v3.14.1), the quality of the assemblies was evaluated using Quast (v5.1) and was annotated using Prokka (v1.14.6; Bankevich et al., 2012; Gurevich et al., 2013; Seemann, 2014). *In silico emm* typing and Multilocus Sequence Typing (MLST) were performed with emm-typer (v.0.2.0) and mlst (v2.11), respectively (Jolley and Maiden, 2010; Microbiological Diagnostic Unit Public Health Laboratory, 2021; Seemann, 2022). The investigation of antibiotic resistance (AMR) genes and virulence factors was carried out with ABRicate (v0.4) by using the Comprehensive Antibiotic Resistance Database (CARD) with 90% coverage (–mincov) and 90% identity (–minid) parameters (Jia et al., 2017; Lacey et al., 2020; Seemann, 2020; Alcolea-Medina et al., 2023). Virulence factors were investigated with a combination of ABRicate (v0.4) by using the Virulence Factor Database (VFDB; Chen et al., 2016) and a BLAST search for the main virulence factors described for *S. pyogenes* in literature (namely, *fbaa, fbp54, fct*A, *fct*B, *grab, has*A, *has*B, *has*C, *hyl*A, *ide*S, *lep*A, *lmb, prtf2, sag*A, *scp*A/*scp*B, *sda1, sda2, sdn, sfb1, sfb*X, *sic, ska, sla, slo, sme*Z, *sof*, *spe*A, *spe*B, *spe*C, *spe*G, *spe*H, *spe*I, *spe*J, *spe*K, *spe*L, *spe*M, *spe*Q, *spe*R, *spd, spd1, spd3, srt*C1, and *ssa*), with 70% coverage and 70% identity parameters (Lacey et al., 2020). The presence of intact bacteriophages was investigated using geNomad (Camargo et al., 2023) and manually curated by searching the identified sequences with the Microbial Nucleotide BLAST (available at https://blast.ncbi.nlm.nih.gov/Blast.cgi?PAGE_TYPE=BlastSearch&BLAST_SPEC=MicrobialGenomes), using the bacteriophages database, while mobile genetic elements (MGEs) identification and plasmid identification were carried out using the MOB-suite tool (v3.1.4; Robertson and Nash, 2018; Robertson et al., 2020).

### Phylogenetic analyses

Single nucleotide polymorphism (SNP) calling was performed with Snippy (v4.6.0; Seemann, 2015), using the *S. pyogenes* MGAS5005 genome (GenBank accession number NC_007297) as reference, after masking phage regions from the alignment and removing regions predicted as possible recombinogenic regions by Gubbins (v3.2.1; Croucher et al., 2015). Reference strains for the various *emm* types available from the PATRIC database (Wattam et al., 2017) and from the NCBI database (https://www.ncbi.nlm.nih.gov/) were also incorporated. The core SNPs identified relative to MGAS5005 were used to infer phylogenetic relationships among the whole genome sequences by Maximum-Likelihood (ML) using IQTREE (v2.0.6) with 1,000 bootstrap replicates under the best nucleotide substitution model (TVM+F+ASC+R2) determined by ModelFinder (Kalyaanamoorthy et al., 2017; Nguyen et al., 2017). The ML tree was visualized and annotated using iTOL (v6.5.2; Letunic and Bork, 2021).

To more accurately evaluate genetic differences among the most prevalent *emm* types (*emm*1 and *emm*12), SNPs were identified by mapping trimmed reads against the MGAS5005 and MGAS2096 (GenBank accession number CP000261) reference strains, respectively, using Snippy (v4.6.0; Seemann, 2015). Pairwise SNP distances were calculated using snp-dists tool (https://github.com/tseemann/snp-dists) and genetic relationships among the isolates were inferred by Minimum Spanning Trees using Grapetree (v1.5.0; Zhou et al., 2018). Potential transmission chains were defined by the presence of at least three strains clustering together in the ML tree with a bootstrap value of 100% and displaying an SNP distance of <10, consistently with other already published articles on *S. pyogenes* (Metcalf et al., 2022; Xie et al., 2023).

### Statistical analysis

Statistical associations between *emm* types and virulence factors were evaluated with respect to iGAS, severe iGAS and severe outcome by Fisher exact test or Mann-Whitney test, as appropriate. To adjust significance for multiple comparisons and confirm potential associations, a Benjamini-Hochberg correction was applied after a chi square test (SPSS v.28.0.1.1).

### Ethical and regulatory aspects

The study was registered by the Milan Area 2 Ethical Committee (#332_2023) and was conducted in accordance with standards of the Helsinki Declaration. At the two hospitals, informed consent for pseudonymized data processing for future research purposes was provided by all patients at the time of hospital admission, as routine procedure. Specific written informed consent was waived because of the retrospective nature of the analysis.

## Results

### 7-year epidemiological surveillance of laboratory-identified GAS infections in the two centers

**Figure 1:**
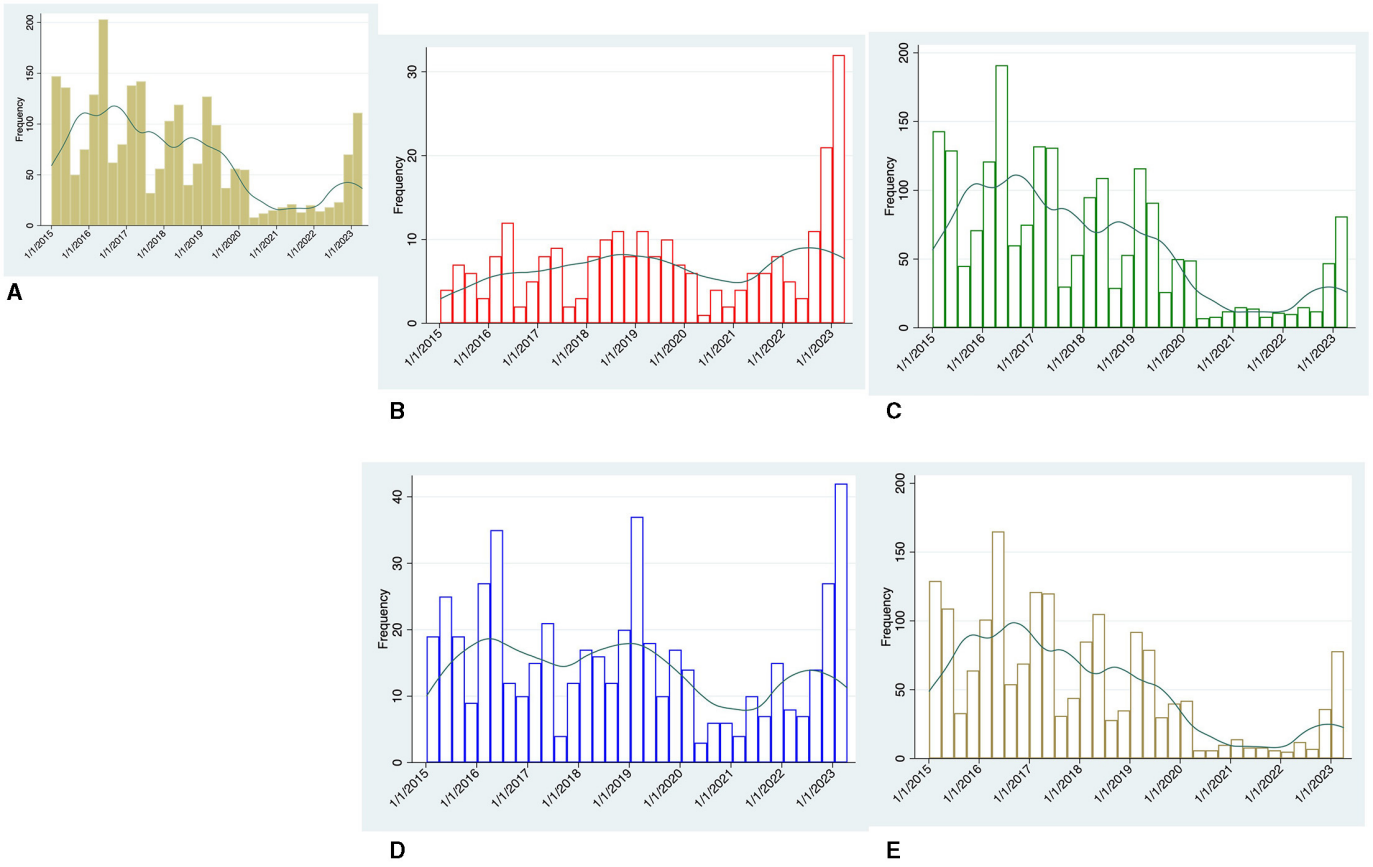
Temporal trend by quarters of microbiologically-confirmed GAS cases between January 2015 and March 2023, Green line represents the kernel density estimation generated for each group. **(A)** Overall cases, **(B)** invasive (iGAS) cases, **(C)** non-iGAS cases, **(D)** adult cases, and **(E)** pediatric cases.

Cases of GAS between January 2015 and March 2023 by quarters are shown in Figure 1, overall (panel A) and stratified according to invasiveness (panels B and C) and age group (panels D and E).

A total of 2,900 cases were identified. Pediatric cases accounted for most of the non-iGAS infections [total cases 2,039: adults 310 (15.2%), children 1,729 (84.8%)], while adult cases accounted for most of the iGAS infections [total cases 251: adults 208 (82.9%), children 43 (17.1%)].

Prior to COVID-19 pandemic, GAS infections peaked in the first two quarters of each year. Unlike non-iGAS infections, the number of iGAS cases was less affected by temporal fluctuations and settled at values permanently below 13 cases every quarter.

During the pandemic years (March 2020 to September 2022), overall GAS cases fell well below the nadir seen in previous years. This trend applies especially to non-iGAS infections, and to a lesser extent to iGAS cases.

Over the last quarter of 2022 and first quarter of 2023 our centers experienced a steep increase in GAS cases. Of note, the largest increase was observed in iGAS infections, which reached 34 cases in the first quarter of 2023 over 3-fold higher than the average case numbers of the same seasonal period in the pre-pandemic years (Figure 1B).

### Description of GAS cases detected during the study period

Between September 2022 and March 2023, 179 laboratory-confirmed GAS infections were identified in the two study hospitals (54 iGAS and 125 non-iGAS). Among them, bacterial strains were available for sequencing in 28 cases. Demographic and clinical features of the study population are detailed in Table 1, Supplementary Table 1.

**Table 1:** Demographic and clinical characteristics and outcomes of the study population.

		***N* = 28**
**Demographics**		
Gender, *n* (%)	Female	8 (28.6)
	Male	20 (71.4)
Age group, *n* (%)	Adults	19 (67.9)
	Children	9 (32.1)
Charlson comorbidity index (age-adjusted), *n* (%)	0	14 (50.0)
	1–3	4 (14.3)
	≥4	10 (35.7)
**Clinical characteristics at presentation**		
Clinical presentation, *n* (%)	URTI	8 (28.6)
	ABSSSI	8 (28.6)
	Arthritis	1 (3.6)
	Fasciitis	7 (25.0)
	Pneumonia	1 (3.6)
	Primary BSI	3 (10.7)
Secondary BSI, *n* (%)	Yes	13 (46.4)
	No	12 (42.9)
	N/A	3 (10.7)
Septic shock, *n* (%)	Yes	9 (32.1)
	No	19 (67.9)
iGAS, *n* (%)	Yes	19 (67.9)
	No	9 (32.1)
Severe iGAS, *n* (%)	Yes	11 (39.3)
	No	17 (60.7)
Antibiotic therapy, *n* (%)	Yes	22 (78.6)
	No	6 (21.4)
Adjunctive therapies, *n* (%)	IVIg	1 (3.6)
	None	27 (96.4)
Source-control, *n* (%)	Yes	8 (28.6)
	No^**^	3 (10.7)
	Not needed	17 (60.7)
**Outcomes**		
ICU admission, *n* (%)	Yes	5 (17.9)
	No	23 (82.1)
In-hospital mortality, *n* (%)	Yes	6 (21.4)
	No	22 (78.6)
Severe clinical outcome^*^, *n* (%)	Yes	9 (32.1)
	No	19 (67.9)

URTI, upper respiratory tract infection; ABSSI, acute bacterial skin and skin structure infection; BSI, bloodstream infection; iGAS, invasive group A streptococcal infection; IVIg, intravenous immunoglobulin; ICU, intensive care unit.

^*^Composite outcome of ICU admission and/or in-hospital mortality.

^**^Deceased before obtaining source-control.

Patients were mainly male (20/28, 71.4%) and adults over 18 years of age (19/28, 67.9%). Median age in adults was 58.5 (Q1–Q3: 44–82) years, in children 5 (Q1–Q3: 3–6) years. Frailty was low on average, with age-adjusted Charlson Comorbidity Index <4 in almost two thirds of cases (18/28, 64.3%). GAS clinical presentation was represented mainly by mild to moderate upper respiratory tract infections (URTI; 8/28, 28.6%), ABSSSI (8/28, 28.6%) and necrotizing fasciitis (7/28, 25.0%). Arthritis was detected in one patient, as was pneumonia (3.6%), while primary bloodstream infections (BSI) without known origin occurred in 3/28 (10.7%) cases. Secondary BSI was diagnosed in almost half of the cohort (13/28, 46.4%), septic shock at presentation in 9/28 (32.1%) cases. Diagnostic criteria of iGAS and severe iGAS infections were met in 19/28 (67.9%) and 11/28 (39.3%) cases, respectively. Five patients (17.9%) required intensive care unit (ICU) admission and 6/28 (21.4%) died during hospitalization. Severe clinical outcome involving ICU admission and/or in-hospital mortality was met in 9/28 (32.1%) cases (Table 1).

Characteristics of patients according to age group are reported in Supplementary Table 1. Pediatric cases were mainly URTI (8/9, 88.9%), while in adults clinical characteristics varied and also presented higher severity, with iGAS and severe iGAS infections diagnosed in 18/19 (94.7%) and 10/19 (52.6%) cases, respectively.

### Bacterial typing and genome characteristics

Whole-genome assemblies of the isolates displayed a median number of contigs of 61 (Q1–Q3: 53–69), with a median N50 of 164,198 bp (Q1–Q3: 130,587–203,753 bp), while the isolates' total genome sizes ranged from 1.75 to 1.93 Mb.

Among the 28 strains, a total of 13 *emm* types were detected, demonstrating the co-circulation of genetically divergent GAS strains in the 8 months analyzed. The most prevalent were *emm*1.0 and *emm*12.0, with 6 strains each (21.4%), followed by *emm*28.0 and *emm*164.2 with 3 strains each (10.7%). Other *emm* types were detected in one strain each (3.6%), and included *emm* types 4.0 (with sub-type 4.19 also observed), 11.0, 22.0, 58.0, 60.1, 82.0, 87.0, 89.0, and 92.0.

**Figure 2:**
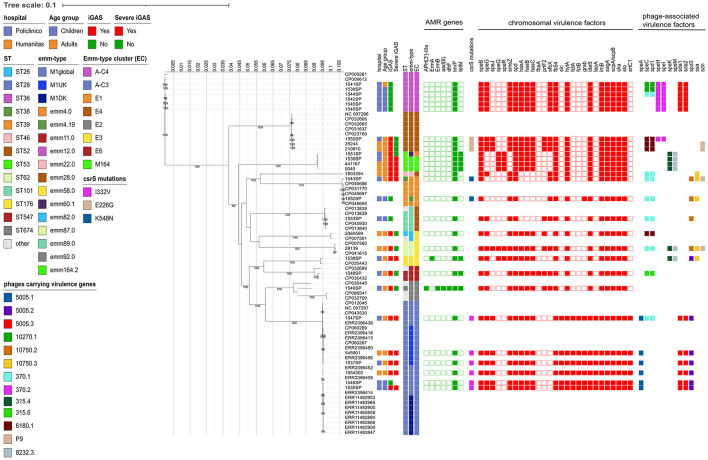
Estimated maximum likelihood phylogenetic analysis of Streptococcus pyogenes isolates (*n* = 28) and reference genomes (*n* = 45). The phylogeny was estimated on a coreSNP of 12,596 bp with IqTree using the best-fit model of nucleotide substitution TVM + F + ASC + R2 with 1,000 replicates fast bootstrapping. Leaves number represent the sample IDs, bootstraps values higher than 90 are shown on branches. Information regarding the samples were reported: hospital, age group, presence of invasive GAS infection (iGAS), presence of severe iGAS, Sequence Type (ST), *emm*-type, *emm*-type cluster (EC), the presence (filled squared) or absence of antimicrobial resistance genes, *csr*S mutations identified, presence (filled square) or absence of virulence factors, divided in chromosomal (red) or phage-associated (color based on phage on which they are present).

By MLST, each ST was found to be unique for each *emm* type, except for ST53, which was found both in strains of *emm* types 60.1 and 164.2 (Figure 2).

When looking at the *emm*-type cluster (EC) classification, which is based on related M proteins sharing similar binding and structural properties, strains mainly belonged to A-C3 (6/28, 21.4%) and A-C4 (6/28, 21.4%), followed by E4 (5/28, 17.9%), E1 (3/27, 10.7%), E3 (3/28, 10.7%), and M164 (3/28, 10.7%).

### Antimicrobial resistance, virulence, and mobile genetic elements

Antimicrobial resistance gene *lmrP* was present in all the GAS strains. In addition, *tet*(M), conferring phenotypic resistance to tetracyclines, was found in 7/28 strains (25.0%, four of them belonging to the 3 *emm*164.2 and *emm*60.1 types), followed by *erm*(A), conferring the Macrolide-Lincosamide-Streptogramin B (MLS_B_) resistance phenotype, found in the *emm*58.0 strain. The *erm*(B) gene, conferring the MLS_B_ resistance phenotype, the genes *aad*(6) and *APH*(3′)-III, conferring resistance to aminoglycosides, and *dfr*F gene, causing resistance to diaminopyrimidine antibiotics, were found together in the *emm* type 92.0 strain (ID 1549SP; Supplementary Table 2). All these data are in line with the phenotypic antimicrobial susceptibility profile of the strains (Supplementary Table 3).

Although the phenotypic test did not detect any large variation in the penicillin resistance, as a full susceptibility was observed for all strains for which the test was available (Supplementary Table 3), mutations at the level of penicillin-binding proteins (PBPs) 1A, 1B, 2A, and 2X were identified (Supplementary Table 4). In particular, the PBP2X mutations included M593T, found in 2/6 *emm*12 strains, mutations I502V, P676S, and K708E, found in the *emm*22 strain, S562T found in the *emm*89 strain and mutations V241I, K213N, and T246A, found in the *emm*58 strain. The M593T mutation was previously found to result in a 2-fold increase in penicillin G and ampicillin MIC when present in combination with the I502V, P676S, and K708E mutations (Yu et al., 2023). However, in our strains this mutation was always present as single PBP2X mutation, probably explaining why no change in the penicillin susceptibility could be observed.

Virulence factors identified were 40 in total, of which 10 present in all strains (Figure 2, Supplementary Table 2). Of the remaining 30, 20 were localized on the chromosome and 10 were phage-associated, and included genes coding for surface proteins, DNases, pilus machinery, capsule synthesis and superantigens (Figure 2, Supplementary Tables 2, 5).

Regarding superantigens, 12 different types could be identified, of which five chromosomally encoded and seven carried by prophages. Among the chromosomal superantigens, *sme*Z was found in all strains (24/28 strains, 85.7%) except for the four strains belonging to *emm*60.1 and 164.2, *spe*G was found in 22 strains (78.6%, absent in strains belonging to *emm* types 60.1, 164.2, 4 and sub-type 4.19), *spe*J was found in 10 strains (35.7%, belonging to *emm* type 28, 87 and 1), and, *spe*Qand *spe*R were always found together in 6 strains (21.4%, belonging to *emm* types 60.1, 164.2, 87 and 58).

The seven prophage-associated superantigens included *spe*A, found in all six strains belonging to *emm*1 (21.4%), *spe*C, found in 14/28 strains always together with DNase *spd1* (50.0%, of which 1/6 *emm*1, 3/6 *emm*12, and 3/3 *emm*28 strains, as well as *emm* types 4, sub-type 4.19, 11, 22, 82, 87, and 92), *spe*H and *spe*I, always found together in 7/28 strains (25.0%, including 6/6 *emm*12 strains and only 1/3 *emm*28 strain), *spe*K and *spe*M, present together in six strains (21.4%, belonging to *emm* types 60.1, 164.2, 87 and 58), and *ssa*, present in 5/28 strains (17.9%, belonging to *emm* types 4 and sub-type 4.19, 22, 58 and 87; Figure 2, Supplementary Table 2). While most of phage-associated superantigens were carried by different phages and *emm* types, in our cohort, *spe*A was always found on the *Streptococcus* phage 5,005.1 (GenBank acc. n. NC_007297.2, positions 983,974–1,022,694) and only in strains belonging to *emm*1.0 (Supplementary Table 5). Similarly, six out of the 7 *spe*H-I (85.7%) were carried by *Streptococcus* phage 370.2 (GenBank acc. n. AE004092.2, positions 778,520–821,004) in the six *emm*12.0 strains (Supplementary Table 5).

Surface proteins identified included fibronectin binding proteins *fbp*54, present in all strains (28/28, 100%), *sfbX*, present in 16/28 strains (57.1%, belonging to *emm* types 4, 11, 12, 22, 28, 87, 89, 92), *prtF2*, present in 8/28 strains (28.6%, belonging to *emm* types 11, 28, 89, 82, 87, and 92), *fbaA*, present only in the *emm*11 strain, and the laminin-binding protein *lmB*, present in all strains (28/28, 100%). Several DNases were also detected, namely *spd*, present in all strains (28/28, 100%), *spd1* in 14 strains (50.0%), *spd3* in 11 strains (39.3%, belonging to *emm* types 1, 4, sub-type 4.19, 58, 89, and 87), *sda1* and *sda2*, always found together in 12/28 strains (42.9%, of which 6/6 *emm*1 and 6/6 *emm*12 strains), and *sdn* in four strains (14.3%, including *emm* type 4, 87 and 2/3 *emm*28 strains). Capsule synthesis genes, *hasA-C*, were absent in four strains, belonging to *emm* types 4, sub-type 4.19, 22, and 89, while grab gene was present only in seven strains, comprising 6/6 *emm*1 strains and the *emm*4.19 sub-type (Figure 2, Supplementary Table 2).

Since the control of several streptococcal virulence factors is mediated by a 2-component regulatory system known as CsrR/CsrS (capsule synthesis regulation), the presence of mutations in the *csrR* and *csrS* genes was evaluated. No *csrR* mutations were identified in any of the 28 strains, whereas three different mutations could be detected in the *csrS* gene. In particular, the I332V mutation was found in all the 6 *emm*1 strains, the E226G mutation in all the 3 *emm*28 strains, and the K548N mutation in the 2 *emm*4 and sub-type 4.19 strains (Figure 2). No N498K mutations, previously found to be associated with invasive infections (Lin et al., 2014), were identified.

Plasmid analysis revealed the presence of 2 non-mobilizable plasmids, both carrying a putative bacteriocin: the pA996 (GenBank acc.n. KC895877) in strain 1549SP (*emm*92.0) and pA852 (acc n. KC895878) in strain 1548SP (*emm*11.0).

### Phylogenetic analysis and genomic characterization of the most common emm types

The ML phylogenetic tree, inferred from a coreSNP alignment of 12,596 bp and constructed including reference genomes available for the *emm* types identified, confirmed the distribution of the strains based on the *emm* type (Figure 2).

Of the 6 strains belonging to *emm*1.0, 4 were from Policlinico Hospital and 2 from Humanitas Hospital. They were characterized by the presence of virulence genes *spe*A, *sic* (Streptococcal inhibitor of complement), *fctA-fctB* (Major pilins), *lep*A (Signal peptidase I), and *srt*C1 (sortase), all absent in the other study strains. The ID 1547SP also carried *the spd1* and *spe*C virulence factors, carried by the same phage (*Streptococcus* phage 370.1, GenBank acc. n. AE004092.2, positions 529,587 to 570,504). As showed by the Minimum Spanning Tree, the median SNP distance characterizing the six strains was 69 (Q1–Q3: 61–83), with no strains displaying a pairwise distance of <10 SNPs, suggesting that they did not belong to a unique transmission chain (Supplementary Figure 1A). By further investigating the genetic diversity, four strains (66.7%) were found to belong to the M1_global_ clone, whereas two strains (namely ID 1537SP, from Policlinico Hospital, and 545,901 from Humanitas Hospital) were found to belong to the M1_UK_ clone, as they carried 27/27 of the clone-defining SNPs. The two M1_UK_ strains differed from each other for 41 SNPs and from the nearest M1_UK_ reference (ERR2396456) by 35 and 20 SNPs, respectively, of which 8 present in both strains (Supplementary material). Among these eight common SNPs, one fell in intergenic regions, three caused missense mutations, and four caused synonymous mutations. The missense mutations were: C423006T, falling in an ABC-2 family transporter; C931062A, falling in an ABC transporter ATP-binding protein; and G1296882A, falling in a Cof-type HAD-IIB family hydrolase (Supplementary material).

The 6 strains belonging to *emm*12.0 exclusively came from Policlinico Hospital and shared the same virulence factors, except for three strains that also carried the phage-associated *spd1* and *spe*C virulence factors (Figure 2). Despite sharing the same hospital origin, the Minimum Spanning Tree strains revealed high diversity among the *6 emm*12.0 strains, which were characterized by a high number of SNPs [median 185 (Q1–Q3: 97–192)], and with the pairwise SNP distance always higher than 10 SNPs, suggesting also in this case that they did not belong to a unique transmission chain (Supplementary Figure 1B, Supplementary material).

Regarding the less prevalent *emm* types, the three strains belonging to *emm*28.0 shared the same virulence factors, with only one strain also carrying the phage associated *spd1*-*spe*C genes. These strains were characterized by high number of SNPs [median 90 (min-max: 11–95)], suggesting once more that they did not belong to a single transmission chain.

The four strains belonging to *emm*164.2 and *emm*60.1 (localized in the same clade in the phylogenetic tree) were characterized by the presence of *tet*M, the same set of chromosomal virulence factors, and the absence of any phage-associated virulence factors. Among these strains, the three belonging to *emm*164.2 were characterized by a low genetic divergence, with a median SNP distance of 6 (min-max: 5–9). On account of their low numbers, it is rather challenging to speculate on potential transmission chains.

### Associations between emm types and virulence factors with age and clinical severity

Table 2 reports the associations between *emm* types and virulence factors with age and clinical severity.

**Table 2:** Associations between *emm* types and virulence factors with age and clinical parameters.

	**Age group**		*P*-value^*^	**iGAS**		*P*-value^*^	**Severe iGAS**		*P*-value^*^
	**Children (*****n*** = **9)**	**Adults (*****n*** = **19)**		**Yes (*****n*** = **19)**	**No (*****n*** = **9)**		**Yes (*****n*** = **11)**	**No (*****n*** = **8)**	
***Emm*** **type**									
1.0 (*n* = 6)	2 (22.2)	4 (21.1)	1.000	5 (26.3)	1 (11.1)	0.630	5 (45.5)	0 (0.0)	**0.045**
12.0 (*n* = 6)	5 (55.6)	1 (5.3)	**0.007**	0 (0.0)	6 (66.7)	**0.0002** ^**#**^	-	-	
28.0 (*n* = 3)	0 (0.0)	3 (15.8)	0.530	3 (15.8)	0 (0.0)	0.530	0 (0.0)	3 (37.5)	0.058
164.2 (*n* = 3)	0 (0.0)	3 (15.8)	0.530	3 (15.8)	0 (0.0)	0.530	3 (27.3)	0 (0.0)	0.228
Others (*n* = 10)^§^	2 (22.2)	8 (42.1)	0.417	8 (42.1)	2 (10.5)	0.417	3 (27.3)	5 (62.5)	0.181
**Virulence factors**									
*fba*A (*n* = 1)	0 (0)	1 (5.3)	1,000	1 (5.3)	0 (0)	1,000	0 (0)	1 (12.5)	0.421
*grab* (*n* = 7)	3 (33.3)	4 (21.1)	0.646	5 (26.3)	2 (22.2)	1.000	5 (45.5)	0 (0.0)	**0.045**
*has*A-C (*n* = 24)	7 (77.8)	17 (89.5)	0.574	17 (89.5)	7 (77.8)	0.574	9 (81.8)	8 (100)	0.485
*prt*F2 (*n* = 8)	1 (11.1)	7 (36.8)	0.214	7 (36.8)	1 (11.1)	0.214	0 (0)	7 (87.5)	**0.0002** ^**#**^
*sda1* + *sda2* (*n* = 12)	7 (77.8)	5 (26.3)	**0.017**	5 (26.3)	7 (77.8)	**0.017**	5 (45.5)	0 (0)	**0.045**
*sdn* (*n* = 4)	0 (0)	4 (21.1)	0.273	4 (21.1)	0 (0)	0.273	1 (9.1)	3 (37.5)	0.262
*sfb*X (*n* = 16)	7 (77.8)	9 (47.4)	0.233	8 (42.1)	8 (88.9)	**0.039**	2 (18.2)	6 (75.0)	**0.024**
*sme*Z (*n* = 24)	9 (100)	15 (78.9)	0.273	15 (78.9)	9 (100)	0.273	8 (72.7)	7 (87.5)	0.603
*spd1* + *spe*C (*n* = 14)	4 (44.4)	10 (52.6)	1.000	9 (47.4)	5 (55.6)	1.000	3 (27.3)	6 (75.0)	0.070
*spd3* (*n* = 11)	4 (44.4)	7 (36.8)	1.000	8 (42.1)	3 (33.3)	1.000	7 (63.6)	1 (12.5)	0.059
*spe*A^**^ (*n* = 6)	2 (22.2)	4 (21.1)	1,000	5 (26.3)	1 (11.1)	0.630	5 (45.5)	0 (0)	**0.045**
*spe*G (*n* = 22)	8 (88.9)	14 (73.7)	0.630	14 (73.7)	8 (88.9)	0.630	7 (63.6)	7 (87.5)	0.243
*spe*H-I (*n* = 7)	5 (55.6)	2 (10.5)	**0.020**	1 (5.3)	6 (66.7)	**0.001** ^**#**^	0 (0.0)	1 (12.5)	0.421
*spe*J (*n* = 10)	2 (22.2)	8 (42.1)	0.417	9 (47.4)	1 (11.1)	0.098	5 (45.5)	4 (50.0)	1.000
*spe*K + *spe*M + *spe*Q + *spe*R (*n* = 6)	0 (0)	6 (31.6)	0.136	6 (31.6)	0 (0)	0.136	4 (36.4)	2 (25.0)	0.659
*ssa* (*n* = 5)	1 (11.1)	4 (21.1)	1.000	4 (21.1)	1 (11.1)	1.000	3 (27.3)	1 (12.5)	0.603
**Median (IQR) n** **°** **of virulence factors**	17 (17–19)	17 (15–20)	0.629	17 (15–24)	17 (17–19)	0.847	17 (13–24)	18 (16–19)	0.963

^*^Associations between emm types or virulence factors with age and clinical outcome were defined by Fisher exact test (two-sided p-value). Associations between number of virulence factors and age or clinical outcome were defined by Mann-Withney test.

^**^Present only in emm1.0, together with fctA, fctB, lepA, sic, and srtC1.

^§^Others included the emm types 4.0 (n = 1), 4.19 (n = 1), 11.0 (n = 1), 22.0 (n = 1), 58.0 (n = 1), 60.1 (n = 1), 82.0 (n = 1), 87.0 (n = 1), 89.0 (n = 1), and 92.0 (n = 1).

^#^p-values that remain significant even after the Benjamini-Hochberg correction. Bold values are statistically significant *p*-value (<0.05).

No significant associations between *emm* type and age group were observed, with the exception of a higher prevalence of *emm*12 infections among pediatric cases [adults 1/19 (5.3%), children 5/9 (55.6%), *P* = 0.007, although not confirmed after the Benjamini-Hochberg correction for multiple comparisons was applied]. iGAS presentation was never found in *emm*12.0 strains, which were found to almost exclusively carry the phage-associated *spe*H-I, virulence factors found to be negatively associated with iGAS (*p* = 0.0002). Of note, this association was confirmed also after the Benjamini-Hochberg correction.

A higher number of severe iGAS cases were detected from strains belonging to *emm*1.0 strains (*P* = 0.045, although not confirmed after the Benjamini-Hochberg correction).

Only one virulence factor, namely *prt*F2, was found to be negatively associated with severe iGAS (*P* = 0.0002, confirmed also after the Benjamini-Hochberg correction), but not to iGAS (*P* = 0.214).

All the associations found between *emm* types and virulence factors with severe iGAS infections were confirmed with severe clinical outcome (data not shown), defined as ICU admission and/or in-hospital mortality.

No significant associations were found between the median number of virulence factors and clinical parameters.

## Discussion

This study describes the increased number of GAS infections in two university hospitals in Milan, Italy, in the last quarter of 2022 and first quarter of 2023, during the surge of invasive cases reported across the European Region (de Gier et al., 2019, 2023; ECDC, 2022; GOV.UK, 2022; WHO, 2022; Guy et al., 2023; Lassoued et al., 2023). We performed genomic characterization of the available GAS strains and investigated relatedness and transmission chains, and the potential association of bacterial genomic characteristics with clinical severity.

Over the last few months, several studies have reported an increase of GAS and iGAS infections starting from early 2022 and mainly affecting the pediatric population. This surge in infections may be related to decreased exposure to GAS and common respiratory viral infection during the COVID-19 pandemic due to physical distancing, which may have led to lower levels of immunity (GOV.UK, 2022). After 2 years of low incidence, an early start of the GAS infection season coupled with high circulating rates of respiratory viruses, which may increase the risk of iGAS diseases (de Gier et al., 2019), is likely to have amplified the resurgence of GAS infections (Lynskey et al., 2019; ECDC, 2022; WHO, 2022; de Gier et al., 2023; Guy et al., 2023; Lassoued et al., 2023).

In our centers, we also observed a sharp increase of GAS and iGAS cases starting from the last quarter of 2022. In contrast to other cohorts, severe infections occurred mostly in adults (almost 95% of iGAS cases) with necrotizing fasciitis and deep ABSSSI with bacteremia as major manifestations. In more than half of cases these represented life-threatening infections (severe iGAS), and mortality occurred in over a quarter of iGAS cases during hospital stay.

Clusters of iGAS infections in adults have been described, mostly found to be caused by outbreaks of specific *emm*-types in hospitals or long-term care facilities (Chalker et al., 2016; Coelho et al., 2019; Trell et al., 2020) or among at-risk population such as homeless people or intravenous drug users (Cornick et al., 2017; Bubba et al., 2019). Our cohort differed for epidemiological characteristics and was not predominated by the clonal expansion of a single *emm*-type, as confirmed by the fact that the 28 strains belonged to 13 different *emm* types and, in the majority of cases, were characterized by a distance higher than 10 SNPs.

Among the detected *emm* types, the most prevalent were *emm*1.0 and *emm*12.0, with 6 strains each. The same *emm* types were found to be the most prevalent in a recently described outbreak in London (Alcolea-Medina et al., 2023), where *emm*1.0 strains were found to cause a higher proportion of invasive cases and predominantly to affect children. Nevertheless, our results showed some differences. First, even though *emm*1.0 strains caused over a quarter of iGAS infections (26.3%) and almost half of all severe iGAS infections (45.5%), they were mostly found in adults. Second, in the London outbreak, 7/9 *emm*1.0 strains were found to belong to the M1_UK_ clone, an *emm*1.0 sublineage characterized by increased SpeA toxin production, while in our samples we found only 2/6 M1_UK_ clone, suggesting a higher differentiation of M1 lineage in our samples. In line with these findings, although M1_UK_ has been described as the predominant clone in *emm*1.0 strains (Demczuk et al., 2019; Li et al., 2020a; Davies et al., 2023), in the UK as well as in other European countries (Rümke et al., 2020; van der Putten et al., 2023; Zhi et al., 2023), recent evidence from Denmark suggested additional differentiation of the M1 lineage with the rising of new M1 clones (Johannesen et al., 2023). In line with that observed in Denmark (Johannesen et al., 2023), the *emm*12.0, the second most prevalent *emm* type identified in our study, mostly detected in children, was found to be negatively associated to invasive infections.

When looking at potential transmission chains in the two most prevalent *emm* types, we found that for both the *emm*1 and *emm*12 strains investigated, strains differed within an *emm* type by more than 10 core SNPs, indicating that they were not linked by recent direct transmission events, and were not likely part of a common transmission chain. When looking at the pairwise SNP distance also in the *emm*28 and *emm*164.2 types, found in three individuals each, only *emm*164.2 showed a limited genetic divergence. Due to the low number of *emm*164.2 strains detected, we cannot speculate about the existence of a real transmission chain, even if all the three strains belonged to males, aged from 57 to 101, affected by severe iGAS, and hospitalized for comorbidities in Humanitas (*n* = 2) and Policlinico (*n* = 1) hospitals. Thus, consistent with studies conducted after COVID-19 pandemic in the pediatric population (de Gier et al., 2023; Guy et al., 2023), the increased incidence of iGAS cases, which we observed predominantly in adult patients, mainly reflects a high circulating rate of several GAS strains in the community rather than direct transmission chains among patients. Understanding and differentiating between these two scenarios might reflect also in the different management of patients, supporting the role of rapid and accurate identification and handling of suspected cases during seasonal epidemics, before enhancing the infection control measures proposed in previous iGAS clusters, such as the screening and treatment of healthcare workers and household contacts (Chalker et al., 2016; Coelho et al., 2019).

As for GAS virulence factors, our findings were in line with what has already been observed in literature, since we found a correlation between *emm* type and the presence of specific superantigen genes. In particular, *spe*A was identified only in the 6 *emm*1.0 strains (Lynskey et al., 2019; Davies et al., 2023), and *spe*H and *spe*I almost exclusively in the 6 *emm*12.0 strains (Li et al., 2020b). As already reported, *emm*12.0 was predominantly found in the pediatric population (Imöhl et al., 2017), while *emm*1.0, and the virulence factors identified in these strains, were found to be associated to severe iGAS infections (Lynskey et al., 2019).

The major limitation of our study is that it included only a subgroup (28/179) of GAS isolated in the two hospitals within the study period. This is due to the retrospective nature of the study and the absence of an active biobanking of all GAS strains. Nevertheless, the majority of missing cases belongs to non-iGAS infections, especially in children. This is due to the characteristics of the two centers (Humanitas does not have a pediatric department) and to the fact that these infections are often diagnosed with rapid antigenic tests without the obtainment of the bacterial isolate (e.g., upper airway respiratory infections, scarlet fever). Moreover, genotyped strains corresponded predominantly to invasive infections (19/28). For this reason, although the results might consistently describe the strains responsible for clinically severe cases in the two hospitals, the data obtained may not be very representative of the circulation of *S. pyogenes* strains in the community at large. Therefore, this could have definitely impacted the ability to detect transmission chains. As further limitation, the modest sample size of our study and the predominance of iGAS infections do not permit us to comprehensively discuss the significant correlations between *emm*-types and clinical manifestations, or to perform other comparisons between patient subsets. Extending the study period and increasing the sample availability are therefore necessary in order to draw definitive conclusions. Nevertheless, we were able to confirm previous evidence on *emm*1.0 and *spe*A correlation with severe iGAS cases (Lynskey et al., 2019). This opens interesting perspectives on the employment of bacterial genome sequencing in clinical practice for clinical risk stratification and patients' management, besides its use for outbreak characterization.

In conclusion, through the integration of genome analysis with clinical and epidemiological patient data, our retrospective study provides a preliminary characterization of iGAS infections that we observed during the surge of invasive cases reported globally. Our results describe the predominant involvement of adult patients in infections requiring hospitalization and give insights into bacterial typing and genomic diversity of the isolates. Integrated genomic studies such as the present emphasize the crucial need to apply genomic surveillance in clinical practice, in order to provide valuable insights on the genomic and evolution of pathogens, enable identification and tracking of emerging strains, and implement targeted infection control measures. In fact, integrating classical epidemiological studies with genomic investigation allowed us to exclude transmission chains during GAS outbreak, helping in ensuring the appropriate infection control strategies and in properly managing severe infections.

## Data availability statement

The datasets presented in this study can be found in online repositories. The names of the repository/repositories and accession number(s) can be found at: https://www.ebi.ac.uk/ena, PRJEB63359.

## Ethics statement

The studies involving humans were approved by Milan Area 2 Ethical Committee (#332_2023). The studies were conducted in accordance with the local legislation and institutional requirements. Written informed consent for participation was not required from the participants or the participants' legal guardians/next of kin in accordance with the national legislation and institutional requirements.

## Author contributions

DM: Conceptualization, Methodology, Writing – original draft. VF: Conceptualization, Formal analysis, Methodology, Writing – original draft. PS: Data curation, Writing – original draft. AL: Conceptualization, Formal analysis, Methodology, Writing – review & editing. LB: Writing – review & editing, Validation. FC: Data curation, Formal analysis, Writing – review & editing. LC: Conceptualization, Data curation, Writing – review & editing. AC: Conceptualization, Methodology, Writing – review & editing. CM: Writing – review & editing. AM: Writing – review & editing. AT: Conceptualization, Data curation, Writing – review & editing. LT: Data curation, Writing – review & editing. VC: Data curation, Writing – review & editing. SC: Writing – review & editing. MB: Writing – review & editing. CA: Conceptualization, Methodology, Supervision, Writing – review & editing. AB: Conceptualization, Methodology, Supervision, Writing – review & editing.
