# Supplementary material for: Increase in invasive group A streptococcal infections in Milan, Italy: a genomic and clinical characterization
Source: Front Microbiol. 2024 Jan 11;14:1287522. doi: 10.3389/fmicb.2023.1287522 (PMC10808429; doi:10.3389/fmicb.2023.1287522)
Supplement: Supplementary file 1 [file Table_1.docx]

**SUPPLEMENTARY TABLES**

**Supplementary Table 1.** Characteristics of enrolled patients according to age group.

|  | | **Adults (N=19)** | **Children (N=9)** |
| --- | --- | --- | --- |
| Demographic | |  |  |
| Gender, n (%) | F | 4 (21.1) | 4 (44.4) |
|  | M | 15 (78.9) | 5 (55.6) |
| Age, median (IQR) | | 58.5 (44,82) | 5 (3,6) |
| Clinical characteristics at presentation | |  |  |
| Clinical presentation, n (%) | URTI | 0 | 8 (88.9) |
|  | ABSSSI | 8 (42.1) | 0 |
|  | Arthritis | 1 (5.3) | 0 |
|  | Fascitis | 6 (31.6) | 1 (11.1) |
|  | Pneumonia | 1 (5.3) |  |
|  | Primary BSI | 3 (15.8) | 0 |
| Polimicrobial infection, n (%) | Yes | 3 (15.8) | 2 (22.2) |
|  | No | 16 (84.2) | 7 (77.8) |
| Secondary BSI, n (%) | Yes | 14 (73.7) | 0 |
|  | No | 3 (15.8) | 9 (100) |
|  | N/A | 3 (15.8) | 0 |
| Septic shock, n (%) | Yes | 8 (42.1) | 1 (11.1) |
|  | No | 11 (57.9) | 8 (88.9) |
| iGAS, n (%) | Yes | 18 (94.7) | 1 (11.1) |
|  | No | 1 (5.3) | 8 (88.9) |
| Severe iGAS, n (%) | Yes | 10 (52.6) | 1 (11.1) |
|  | No | 9 (47.4) | 8 (88.9) |
| Antibiotic therapy, n (%) | Yes | 19 (100) | 3 (33.3) |
|  | No | 0 | 6 (66.7) |
| Source-control, n (%) | Yes | 7 (36.8) | 1 (11.1) |
|  | No** | 3 (15.8) | 0 |
|  | Not needed | 9 (47.4) | 8 (88.9) |
| Outcomes |  |  |  |
| ICU admission, n (%) | Yes | 4 (21.1) | 1 (11.1) |
|  | No | 15 (78.9) | 8 (88.9) |
| In-hospital mortality, n (%) | Yes | 6 (31.6) | 0 |
|  | No | 13 (68.4) | 9 (100) |
| Severe clinical outcome*, n (%) | Yes | 8 (42.1) | 1 (11.1) |
|  | No | 11 (57.9) | 8 (88.9) |
| Antibiotic therapy |  |  |  |
| ABT, n (%) | No | 0 | 6 (66.7) |
|  | Yes | 19 (100) | 3 (33.3) |
| Empirical ABT duration, mean (SD) | | 2 (2) | 8 (2) |
| Tailored ABT duration, mean (SD) | | 18 (10) | 29*** |
| Overall ABT duration, mean (SD) | | 20 (11) | 18 (15) |

ABT: antibiotic therapy; URTI: upper respiratory tract infection; ABSSI: acute bacterial skin and skin structure infection; BSI: bloodstream infection; iGAS: invasive group A streptococcal infection.

*Composite outcome of ICU admission and/or in-hospital mortality; **deceased before obtaining source-control; **one case only

**Supplementary Table 2.** Antimicrobial resistance genes and virulence factors identified in the 28 GAS strains.

| **Gene** | **Name** | **Function** | **N(%)** |
| --- | --- | --- | --- |
| **Antimicrobial resistance genes** | | | |
| *APH*(3’)-IIIa | aminoglycoside phosphotransferase | Aminoglycoside resistance | 1(3.6)* |
| *erm*A | Erm 23S ribosomal RNA methyltransferase | Streptogamin, macrolide, lincosamide resistance | 1(3.6)** |
| *erm*B | Erm 23S ribosomal RNA methyltransferase | Streptogamin, macrolide, lincosamide resistance | 1(3.6)* |
| *aad*(6) | aminoglycoside nucleotidyltransferase | Aminoglycoside resistance | 1(3.6)* |
| *dfr*F | trimethoprim resistant dihydrofolate reductase dfr | Diaminopyrimidine resistance | 1(3.6)* |
| *lmr*P | major facilitator superfamily (MFS) | Lincosamide, macrolide, streptogamin, tetracycline resistance | 28(100) |
| *tet*M | tetracycline-resistant ribosomal protection protein | Tetracycline resistance | 7(25.0)*** |
| **Virulence factors present in all strains** | | | |
| *fbp*54 | fibronectin-binding protein 54 | Surface protein | 28(100) |
| *hyl*A | Hyaluronidase | Extracellular hyaluronidase | 28(100) |
| *ide*S | IgG-degrading enzyme | Secreted protein, CD11b homolog | 28(100) |
| *lmB* | Laminin-binding protein | Adherence function | 28(100) |
| *spd* | DNase B, also known as mitogenic factor | DNase | 28(100) |
| *sag*A | Streptolysin S associated protein | Streptolysin S toxin precursor | 28(100) |
| *scp*A/*scp*B | streptococcal C5a peptidase | Multidomain cell-envelope subtilase that cleaves complement component C5a | 28(100) |
| *ska* | Streptokinase A precursor | Streptokinase, induces inflammation by complement activation | 28(100) |
| *slo* | Streptolysin O | Oxygen-labile streptolysin O | 28(100) |
| *spe*B | Streptococcal pyrogenic exotoxin B | Present in all *S. pyogenes*, Integrin-binding cysteine protease. | 28(100) |
| **Virulence factors present in a subset of strains** | | | |
| *fba*A | Fibronectin-binding protein of group A streptococci type A | Surface protein | 1(3.6) |
| *fct*A | Major pilin FctA | Pilus assembly | 6(22.2) |
| *fct*B | Minor pilin FctB | Pilus assembly | 6(22.2) |
| *grab* | G-related α2macroglobulin-binding protein | Surface protein. High affinity for α2-macroglobulin (α2M) | 7(25.9) |
| *has*A | Hyaluronan synthase HasA | Glycosaminoglycan synthesis (capsule) | 24(85.7) |
| *has*B | UDP-glucose 6-dehydrogenase HasB | Catalyzes the formation of UDP-glucuronic acid which is required for capsular hyaluronic acid synthesis | 24(85.7) |
| *has*C | UTP--glucose-1-phosphate uridylyltransferase HasC | The reaction catalyzed by the hasC product yields a substrate for UDP-glucose dehydrogenase encoded by hasB, whose reaction product is, in turn, a substrate for hyaluronan synthase encoded by hasA (capsule) | 24(85.7) |
| *lep*A | Signal peptidase I | FCT-form specific chaperone, required for pilus assembly | 6(22.2) |
| *prtF2* | *S. pyogenes* fibronectin binding protein | Surface protein | 8(28.6) |
| *sda1* | streptodornase D 1 | DNAse | 12(42.9) |
| *sda2* | streptodornase D 2 | DNAse | 12(42.9) |
| *sdn* | streptodornase | DNAse | 4(14.3) |
| *sfbX* | *S. pyogenes* fibronectin binding protein | Surface protein | 16(57.1) |
| *sic* | Streptococcal inhibitor of complement-mediated lysis | Hypervariable extracellular protein | 6(22.2) |
| *sme*Z | Streptococcal mitogenic exotoxin Z | Superantigen, associated with streptococcal toxic shock syndrome and streptococcal scarlet fever | 24(85.7) |
| *spd1* | Streptococcal DNAse | DNase | 14(50.0) |
| *spd*3 | Streptococcal DNAse | Dnase | 11(39.3) |
| *spe*A | streptococcal exotoxin A | Superantigen, | 6(22.2) |
| *spe*C | streptococcal exotoxin C | Superantigen, | 14(50.0) |
| *spe*G | Streptococcal exotoxin G | Superantigen, | 22(81.5) |
| *spe*H | streptococcal exotoxin H | Superantigen, | 7(25.9) |
| *spe*I | streptococcal exotoxin I | Superantigen, | 7(25.9) |
| *spe*J | streptococcal exotoxin J | Superantigen | 10(35.7) |
| *speK* | streptococcal exotoxin K | Superantigen, | 6(22.2) |
| *speM* | streptococcal exotoxin M | Superantigen, | 6(22.2) |
| *speQ* | streptococcal exotoxin Q | Superantigen, | 6(22.2) |
| *speR* | streptococcal exotoxin R | Superantigen, | 6(22.2) |
|  |  |  |  |
| *srt*C1 | Sortase | Pilus polymerase machinery | 6(22.2) |
| *ssa* | streptococcal superantigen SSA | Phage-associated superantigen, associated with streptococcal toxic shock syndrome and streptococcal scarlet fever | 5(17.9) |

* these genes are present in the *emm* type 92.0 strain 1549SP

** gene present in the *emm* type 58.0 strain 1538SP

*** gene present in emm164.2: 3 strains; *emm*11.0: 1 strain; *emm*58.0: 1 strain; *emm*60.1: 1 strain; *emm*92.0: 1 strain

**Supplementary table 3.** Antimicrobial susceptibility profile of the isolates and overview of the antibiotic therapy employed.

|  | | **N=28** |
| --- | --- | --- |
| GAS antimicrobial susceptibility profile |  |  |
| Penicillin sensitivity, n (%) | N/A | 1 (3.6) |
|  | S | 27 (96.4) |
| Clindamycin sensitivity, n (%) | N/A | 4 (14.3) |
|  | R | 2 (7.1) |
|  | S | 22 (78.6) |
| Erythromycin sensitivity, n (%) | N/A | 1 (3.6) |
|  | R | 2 (7.1) |
|  | S | 25 (89.3) |
| Tetracycline sensitivity, n (%) | N/A | 4 (14.3) |
|  | R | 7 (25.0) |
|  | S | 17 (60.7) |
| Bacterial co-infections/superinfections |  |  |
| bacterial co-infection at presentation | Yes | 5 (17.9) |
|  | No | 23 (82.1) |
| bacterial superinfections during hospitalization, n (%) | Yes | 4 (14.3) |
|  | No | 24 (85.7) |
| Antibiotic therapy |  |  |
| ABT, n (%) | Yes | 22 (78.6) |
|  | No | 6 (21.4) |
| Empirical ABT, n (%) | AMC | 1 (3.6) |
|  | CPR | 1 (3.6) |
|  | CRO | 2 (7.1) |
|  | CRO + CLI | 1 (3.6) |
|  | CRO + CLI + DAP | 1 (3.6) |
|  | CRO + DAP | 1 (3.6) |
|  | CRO + VAN | 1 (3.6) |
|  | SAM + DAP | 1 (3.6) |
|  | TZP + DAP | 1 (3.6) |
|  | TZP + VAN + CLI | 1 (3.6) |
|  | TZP | 5 (17.9) |
|  | TZP + DAP + CLI | 5 (17.9) |
|  | MEM + VAN + CLI | 1 (3.6) |
|  | none | 6 (21.4) |
| Tailored ABT, n (%) | PEN G + CLI | 5 (17.9) |
|  | AMC + CLI | 2 (7.1) |
|  | CRO | 2 (7.1) |
|  | CRO + CLI | 3 (10.7) |
|  | CRO + LZD | 1 (3.6) |
|  | TZP + CLI | 2 (7.1) |
|  | TZP + DAP + CLI | 1 (3.6) |
|  | LZD | 1 (3.6) |
|  | none/unchanged empirical ABT | 11 (39.3) |
| Time from clinical onset to empirical ABT start (days), mean (SD) | | 1 (1) |
| Duration of empirical ABT (days), mean (SD) | | 3 (3) |
| Time from clinical onset to targeted ABT start (days), mean (SD) | | 4 (3) |
| Duration of tailored ABT (days), mean (SD) | | 18 (10) |
| Overall duration of ABT (days), mean (SD) | | 21 (11) |

ABT: antibiotic therapy; AMC: amoxicillin/clavulanate; CPR: cefprozil; CRO: ceftriaxone; CLI: clindamycin; DAP: daptomycin; VAN: vancomycin; MEM: meropenem; AMK: amikacin; SAM: ampicillin/sulbactam; TZP: piperacillin/tazobactam; LZD: linezolid; PEN G: penicillin G.

**Supplementary Table 4. Deduced amino acid substitutions in the Penicillin-Binding Proteins (PBPs) of the 28 *S. pyogenes* isolates**

| **ID** | **Emm-type** | ***pbp*1A** | ***pbp*1B** | ***pbp*2A** | ***pbp*2x** |
| --- | --- | --- | --- | --- | --- |
| 5045 | 164.2 | D321N, A556T | I134V, F279Y, G308R | T27A, N43H, T59I, R90H | - |
| 25244 | 28 | T699I, G709S, S710N | I134V, F279Y, V434I, P688S | T59I, R90H | - |
| 28139 | 87 | - | I134V, F279Y, K289E | T27A, T59I, R90H | - |
| 210810 | 28 | T699I, G709S, S710N | I134V, F279Y, V434I, P688S | T59I, R90H | - |
| 441167 | 164.2 | D321N, A556T | I134V, F279Y, G308R | T27A, T59I, R90H | - |
| 545901 | 1 | - | - | - | - |
| 1554302 | 1 | - | - | - | - |
| 1903394 | 22 | I39V, D519N | I134V, F279Y, V541I | T27A, T59I, R90H | I502V, P676S, K708E |
| 2060599 | 82 | Q263R, T699I, G709S, S710N | I134V, F279Y | T59I, R90H | N359S |
| 1535SP | 1 | - | - | - | - |
| 1536SP | 164.2 | D321N, A556T | I134V, F279Y, G308R | T27A, T59I, R90H | - |
| 1537SP | 1 | - | - | - | - |
| 1538SP | 58 | T91A, T303A, N707T | I134V, F279Y | T59I, R90H, N582K | V24I, K213N, T246A |
| 1539SP | 12 | T688M, T699I, G709S, S710N | I134V, F279Y | T59I, R90H | - |
| 1540SP | 12 | T699I, G709S, S710N | I134V, F279Y | T59I, R90H | M593T |
| 1541SP | 12 | T688M, T699I, G709S, S710N | I134V, F279Y | T59I, R90H | - |
| 1542SP | 12 | T699I, G709S, S710N | I134V, F279Y | T59I, R90H | - |
| 1543SP | 4 | A443T | F279Y, E349D, G390D | T59I, R90H | - |
| 1544SP | 12 | T699I, G709S, S710N | I134V, F279Y | T59I, R90H | - |
| 1545SP | 12 | T699I, G709S, S710N | I134V, F279Y | T59I, R90H | M593T |
| 1546SP | 1 | - | - | - | - |
| 1547SP | 1 | - | - | - | - |
| 1548SP | 11 | - | I134V, F279Y | D56V, T59I, R90H | - |
| 1549SP | 92 | - | I134V, A239S, F279Y | T27A, P40S, A42V, T59I, R90H | - |
| 1550SP | 28 | T699I, G709S, S710N | I134V, F279Y, V434I, P688S | T59I, R90H | - |
| 1551SP | 60.1 | D321N, A556T | I134V, F279Y, G308R | T27A, T59I, R90H | - |
| 1552SP | 4.19 | - | F279Y, E349D, G390D | T59I, R90H | - |
| 1553SP | 89 | G709S, S710N | E130K, I134V, F279Y, V541I | T27A, T59I, R90H | S562T |

SF370 strain (GenBank accession n° AE004092.2) was used as the reference strain. -, no amino acid substitution identified

**Supplementary Table 5**. **Distribution of virulence factors among the identified bacteriophages.**

| **Bacteriophages**  **(GenBank acc .n°, position)** | ***spe*A**  **(n=6)** | ***spe*C-*spd1***  **(n=14)** | ***spe*I*-spe*H**  **(n=7)** | ***spe*K**  **(n=6)** | ***spe*M**  **(n=6)** | ***sda1*-*sda2***  **(n=12)** | ***spd3***  **(n=11)** | ***ssa***  **(n=5)** | ***sdn***  **(n=4)** | **Median (IQR) % identity** |
| --- | --- | --- | --- | --- | --- | --- | --- | --- | --- | --- |
| 5005.1  (NC_007297.2, 983974..1022694) | 6 (100%)* |  |  |  |  |  |  |  |  | 99.9 (99.9-99.9) |
| 5005.2  (NC_007297.2, 1145829..1178945) |  |  |  |  |  |  | 7  (63.6%) |  |  | 99.9 (99.9-99.9) |
| 5005.3  (NC_007297.2, 1385601..1426222) |  |  |  |  |  | 12  (100%) |  |  |  | 99.9 (99.9-99.9) |
| 10270.1  (NC_008022.1, 528592..572664) |  | 2 (14.3%) |  |  |  |  |  |  |  | 99.9, 99.9 |
| 10750.2  (NC_008024.1, 804055..841975) |  |  |  |  |  |  | 4  (36.4%) |  |  | 99.9 (99.6-99.9) |
| 10750.3  (NC_008024.1, 1220659..1256255) |  |  |  |  |  |  |  | 5  (100%) |  | 99.9 (98.9-99.9) |
| 370.1  (AE004092.2, 529587..570504) |  | 7  (50.0%) |  |  |  |  |  |  |  | 98.0 (97.4-98.3) |
| 370.2  (AE004092.2, 778520..821004) |  |  | 7  (100%) |  |  |  |  |  |  | 97.6 (97.2-98.3) |
| 315.4  (NC_004587) |  |  |  | 6  (100%) |  |  |  |  |  | 90.6  (87.5-98.6) |
| 315.6  (NC_004589.1) |  | 1  (7.1%) |  |  |  |  |  |  |  | 95.0 |
| 6180.1  (NC_007296.2, 986244..1032511) |  | 4  (28.6%) |  |  |  |  |  |  |  | 99.9 (99.9-99.9) |
| 8232.3  (AE009949,  1041142..1087883) |  |  |  |  | 6  (100%) |  |  |  |  | 88.5  (81.2-92.9) |
| P9  (NC_009819.1) |  |  |  |  |  |  |  |  | 4  (100%) | 97.7 (95.6- 99.1) |

Numbers and prevalences (in the brackets) of each virulence factor against the encoding phage are reported**.**

Median (IQR) percentage identity for each phage are also reported.

***** this virulence factor was present only in *emm* type 1.0 strains
