# Supplementary figures and images for: Increase in invasive group A streptococcal infections in Milan, Italy: a genomic and clinical characterization
Source: Front Microbiol. 2024 Jan 11;14:1287522. doi: 10.3389/fmicb.2023.1287522 (PMC10808429; doi:10.3389/fmicb.2023.1287522)

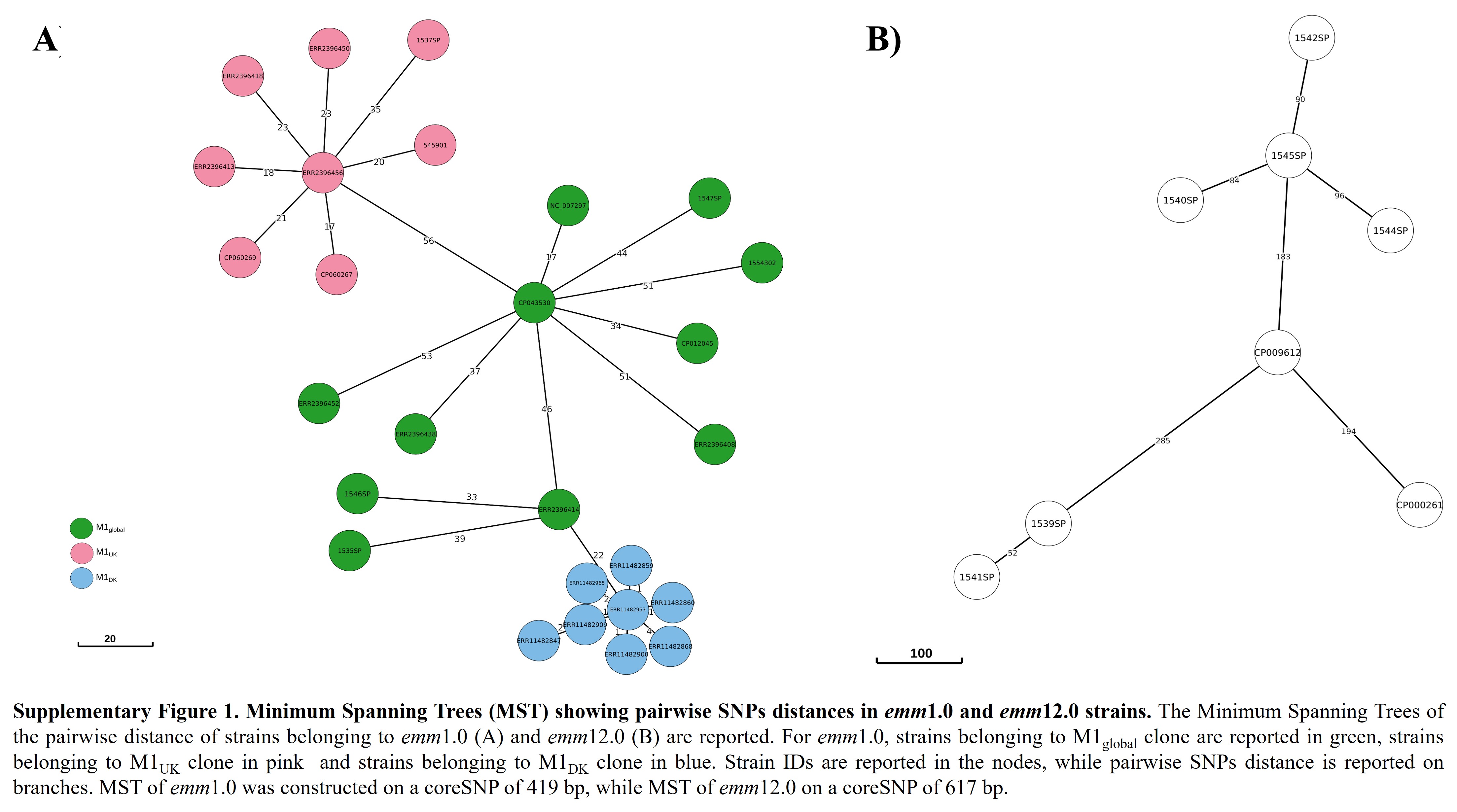

Supplement: Supplementary file 2 [file Image_1.JPEG]
